# Electrostatic complexation of polyelectrolyte and magnetic nanoparticles: from wild clustering to controllable magnetic wires

**DOI:** 10.1186/1556-276X-9-198

**Published:** 2014-05-01

**Authors:** Minhao Yan, Li Qu, Jiangxia Fan, Yong Ren

**Affiliations:** 1 State Key Laboratory Cultivation Base for Nonmetal Composites and Functional Materials, School of Materials Science and Engineering, Southwest University of Science and Technology, Mianyang 621010, China

**Keywords:** *γ*-Fe_2_O_3_ nanoparticles, Polyelectrolytes, Electrostatic complexation, Desalting transition, Magnetic wires

## Abstract

We present the electrostatic complexation between polyelectrolytes and charged nanoparticles. The nanoparticles in solution are *γ*-Fe_2_O_3_ (maghemite) spheres with 8.3 nm diameter and anionic surface charges. The complexation was monitored using three different formulation pathways such as direct mixing, dilution, and dialysis. In the first process, the hybrids were obtained by mixing stock solutions of polymers and nanoparticles. A ‘destabilization state’ with sharp and intense maximum aggregation was found at charges stoichiometry (isoelectric point). While on the two sides of the isoelectric point, ‘long-lived stable clusters state’ (arrested states) were observed. Dilution and dialysis processes were based on controlled desalting kinetics according to methods developed in molecular biology. Under an external magnetic field (*B* = 0.3 T), from dialysis at isoelectric point and at arrested states, cationic polyelectrolytes can ‘paste’ these magnetic nanoparticles (NPs) together to yield irregular aggregates (size of 100 μm) and regular rod-like aggregates, respectively. These straight magnetic wires were fabricated with diameters around 200 nm and lengths comprised between 1 μm and 0.5 mm. The wires can have either positive or negative charges on their surface. After analyzing their orientational behavior under an external rotating field, we also showed that the wires made from different polyelectrolytes have the same magnetic property. The recipe used a wide range of polyelectrolytes thereby enhancing the versatility and applied potentialities of the method. This simple and general approach presents significant perspective for the fabrication of hybrid functional materials.

## Background

Polyelectrolytes (PEs) are defined as polymer chains composed of monomer units having ionizable groups. Their prominent features are a high solubility and strong adsorbing capacity at oppositely charged surfaces. The absorption of PEs on charged colloidal material has been investigated by a range of experimental methods [1-6], theoretical models [7-14], and computer simulations [15-22]. This absorption (or electrostatic complexation) is thought to be at the origin of many fundamental assembly mechanisms [23-27]. These mechanisms were also recognized as essential in several applications, including flocculation of colloidal particles in water treatment [28,29], and complex formation involving DNA in gene therapy and genetic regulation [30-32]. The final structure formed by the adsorption of positively charged histone proteins on a single negatively charged DNA is called chromatin; the DNA is wrapped around the histone core and preserves its helical structure [33]. Moreover, the formation of multilayer PE films and micro- and nanosized capsules by successive layer-by-layer deposition of anionic and cationic PEs at surfaces has received great interest in the past 10 years [34-37].

In fact, the attractive interactions between PEs and oppositely charged colloids are strong, and the direct mixing of solutions containing such entities yields a phase separation. This is the case, e.g., for anionic PEs and cationic surfactants, for which micellar coacervate and liquid crystalline phases have been observed [38-40]. Means to control the electrostatically driven attractions and to preserve the colloidal stability were developed using copolymers and in particular polyelectrolyte-neutral block copolymers [27,41]. These fully hydrosoluble macromolecules were found to co-assemble spontaneously with different types of systems, such as surfactants [42-44], polymers [45,46], and proteins [47], yielding core-shell structures. As a result of the co-assembly, the cores of the aggregates were described as a dense coacervate microphase comprising the oppositely charged species and surrounded by a neutral corona made from the neutral blocks. Thanks to this neutral corona, the attractive interaction can be slowed down and the size of the co-assemblies (the colloidal stability) can be limited at colloidal range. In order to better control their aggregation, a novel mixing protocol for bringing anionic *γ*-Fe_2_O_3_ nanoparticles (NPs) and cationic-neutral diblock copolymers together was elaborated [48]. This protocol was inspired from molecular biology techniques developed for the *in vitro* reconstitutions of chromatin [49]. It consisted first in the screening of the electrostatic interactions by bringing the dispersions to high ionic strength (1 M of inorganic salt), and in a second step in the removal of the salt by dialysis or by dilution. We have applied this ‘desalting kinetic’ method for the fabrication of spherical and rod-like clusters with regular spherical and cylindrical form [48,50,51].

In terms of practical application, we evaluate here the potential generalization of this method to widespread homopolyelectrolytes (homoPEs). For the homoPEs without neutral part, we need to control their strong interaction with oppositely charged NPs and find a stable colloidal cluster states as polyelectrolyte-neutral block copolymers. As a matter of fact, when the PE chains are adsorbed on the particle surface, there exist competing electrostatic interactions which are attractive between the polymer and the oppositely charged surface and repulsive between the same charged polymers. Nguyen and Shklovskii explained that when the surface charge of the particle is reduced by condensed oppositely charged polyions, the correlation-induced short-range attraction dominates the long-range electrostatic repulsion, leading to the cluster formation [52-54]. Close to the isoelectric point, such destabilization (and eventually the precipitation of the solid fraction) is observed [55]. However, symmetrically on both sides of the isoelectric point, the formation of long-lived, finite size aggregates overstays [56-58]. These aggregates have a size ranging from a few hundred nanometers to a few microns, getting closer to the border of the ‘destabilization zone’. They form almost immediately when the polyelectrolyte is added to the colloidal suspension and then remain stable in time for weeks, without showing any tendency toward further aggregation.

Here, we presented complete experimental details and results of the electrostatic complexation between cationic homoPEs and negatively charged superparamagnetic iron oxide NPs. By using direct mixing method, we evidenced their ‘destabilization state’ at charges stoichiometry (isoelectric point) and ‘long-lived stable clusters state’ named arrested states apart of isoelectric point. Then, we applied the ‘desalting kinetic’ method to their complexation in the presence of an externally applied magnetic field (0.3 T). At isoelectric point, large and irregular aggregates with macroscopic sedimentation were obtained. Apart of isoelectric point (at arrested state), regular and elongated magnetic wires can be obtained. By tuning charges ratio, we can also select the overall surface charge (either positive or negative) of these magnetic wires. Moreover, we derive the probability distribution function of wire length and study their mechanisms of reorientations under the application of a magnetic field. The experimental observations lead us to the conclusion that the wires formed with homoPEs are superparamagnetic as well as the wires made from polyelectrolyte-neutral block copolymers.

## Methods

### Building block materials

**Figure 1:**
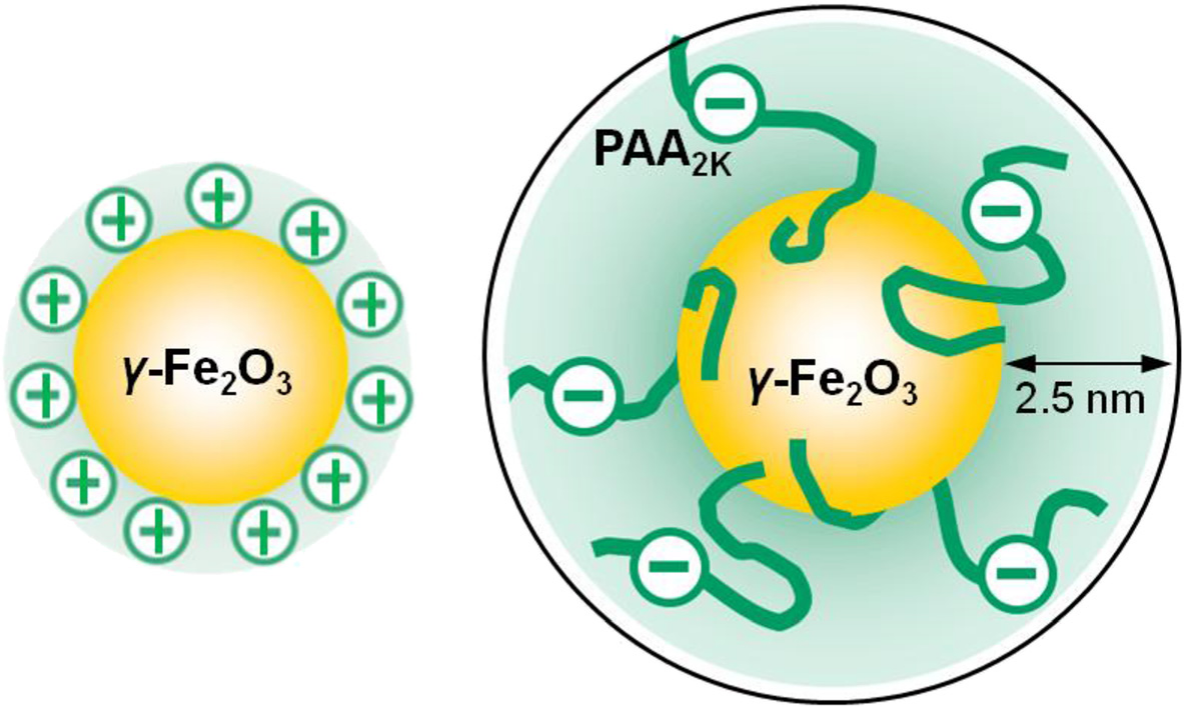
**Schematic description of bare *γ*-Fe**_**2**_**O**_**3**_**nanoparticles (left) and PAA**_**2K **_**polymer coatings around particle (right).** The organic functionalities were adsorbed on the particle surfaces through electrostatic complexation.

The synthesis of the superparamagnetic NPs investigated here was elaborated by Massart et al. using the technique of ‘soft chemistry’ [59]. Based on the polycondensation of metallic salts in alkaline aqueous media, this technique resulted in the formation of magnetite (Fe_3_O_4_) NPs of sizes comprised between 4 and 15 nm. Magnetite crystallites were further oxidized into maghemite (*γ*-Fe_2_O_3_) and sorted according to their size. In the conditions of the synthesis (pH 1.8, weight concentration *c* ~ 10 wt.%), the magnetic dispersions were stabilized by electrostatic interactions arising from the native cationic charges at the surface of the particles. In this work, particles of diameters 8.3 nm were synthesized. The particle size distributions were characterized by vibrating sample magnetometry (VSM), transmission electron microscopy (TEM), and dynamic light scattering (DLS) (see Additional file 1: SI-1). In order to improve their colloidal stability, the cationic particles were further coated by poly(acrylic acid) oligomers with molecular weight 2,000 × *g* mol^−1^ using the precipitation-redispersion process described previously [60]. The hydrodynamic sizes found in *γ*-Fe_2_O_3_-PAA_2K_ dispersions were 5 nm (34 nm) above that of the bare particles (29 nm), indicating the presence of a 2.5-nm PAA_2K_ brush surrounding the particles (see in Figure 1). The fully characterizations of the bare and coated particles was shown in Table 1.

**Table 1:** Characteristics of the particles used in this work

***γ*-Fe**_**2**_**O**_**3**_	
**Characteristics**	**Values**
*D*^VSM^(nm)	8.3
*s*^VSM^	0.26
*D*^TEM^(nm)	9.3
*s*^TEM^	0.18
$M_{W}^{\mathrm{SLS}}\left(g\;{\mathrm{mol}}^{-1}\right)$	5.8 × 10^6^
$M_{W}^{\mathrm{TEM}}\left(g\;{\mathrm{mol}}^{-1}\right)$	3.8 × 10^6^
$D_{H}^{\mathrm{bare}}\left(\mathrm{nm}\right)$	29
$D_{H}^{\mathrm{coated}}\left(\mathrm{nm}\right)$	34
$N_{\mathrm{ads}}^{{\mathrm{PAA}}_{2K}}$	470 ± 30
$N^{{\mathrm{COO}}^{-}}$	12,500 ± 50

Where*D*^VSM^ is the median diameter of the bare particles determined by VSM; *s*^VSM^ is the polydispersity of the size distribution of the bare particles determined by VSM; *D*^TEM^ is the median diameter of the bare particles from TEM; *s*^TEM^is the polydispersity of the size distribution of the bare particles determined by TEM; $M_{W}^{\mathrm{SLS}}$ is the molecular weight of the bare particles derived from static light scattering experiments; $M_{W}^{\mathrm{TEM}}$ is the molecular weight of the bare particles derived from the size distribution measured by TEM; $D_{H}^{\mathrm{bare}}$ is the hydrodynamic diameter of the bare particles, as determined by DLS; $D_{H}^{\mathrm{coated}}$ is the hydrodynamic diameter of the PAA_2K_-coated particles, as determined by DLS; $N_{\mathrm{ads}}^{{\mathrm{PAA}}_{2K}}$ is the number of PAA_2K_polymers adsorbed on the 8.3-nm particles and $N^{{\mathrm{COO}}^{-}}$ is the number of carboxylate groups available at the surface of the particle.

**Figure 2:**
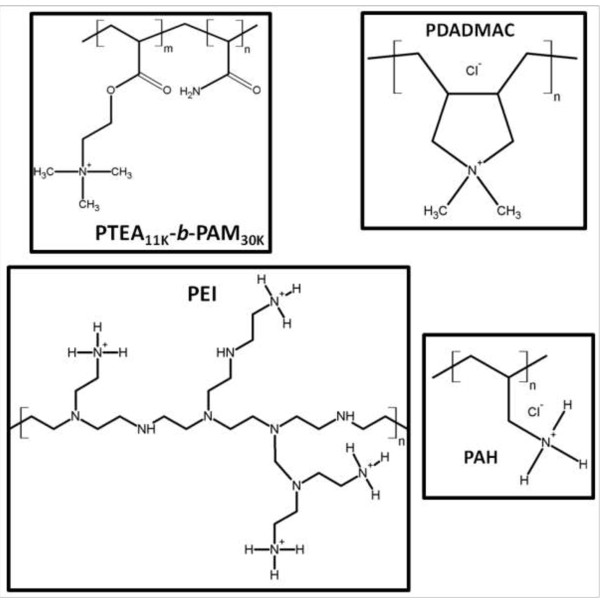
**Molecular structures of PTEA**_**11K**_**-*b*-PAM**_**30K**_**, PDADMAC, PEI, and PAH.**

As reported before, the anionically charged NPs have been co-assembled with a cationic-neutral diblock copolymers [48,50], referred to as poly(trimethylammonium ethylacrylate)-b-poly(acrylamide) (PTEA_11K_-*b*-PAM_30K_, *M*_w_ = 44,400 g mol^−1^). The copolymers were synthesized by MADIX® controlled radical polymerization, which is a Rhodia patented process [61,62]. Light scattering experiment was performed on the copolymer aqueous solutions to determine the weight-averaged molecular weight *M*_w_(44,400 ± 2,000 g mol^−1^) and mean hydrodynamic diameter *D*_H_ (11 nm) of the chains [63]. The molecular weights targeted by the synthesis were 11000-*b*-30000 g mol^−1^, corresponding to 41 monomers of trimethylammonium ethylacrylate methylsulfate and 420 monomers of acrylamide, in fair agreement with the experimental values. In the following, this polymer will be abbreviated as PTEA_11K_-*b*-PAM_30K_[63]. The polydispersity index was determined by size exclusion chromatography at 1.6. Here, we used commercial homoPEs to co-assembly NPs as a comparison with cationic-neutral PEs. Poly(diallyldimethylammonium chloride) (PDADMAC, *M*_w_ = 100,000, 35 wt.% in H_2_O), poly(ethyleneimine) (PEI, *M*_w_ = 2,000, 50 wt.% in H_2_O), and poly(allylamine hychloride) (PAH, *M*_w_ = 15,000) were obtained from Sigma-Aldrich, St. Louis, MO, USA, and used as received. The molecular formulas are given in Figure 2.

### Sample preparation

NPs/PEs aggregates were prepared according to three different methods. The first method, called direct mixing, utilized stock polymer and NPs solutions prepared without added salt. The two other protocols, dilution and dialysis, were based on a principle of desalting processes, starting all runs at the initial ionic strength *I*_*S*_ = 3 M of ammonium chloride (NH_4_Cl). The ionic strength was defined as [64]

(1) $$I_{S}=\frac{1}{2}\sum_{i}c_{i}z_{i}^{2}$$

where *c*_*i*_ and *z*_*i*_ denote the concentration and valency of the ionic atomic species in solution, respectively.

#### Direct mixing

NPs/PEs complexes were obtained by mixing stock solutions prepared at the same weight concentration (*c* ∼ 0.1 wt.%) and same pH (pH 8). The mixing of the two initial solutions was characterized by the particles-polymers charges ratio *Z. Z* is defined as the structural charges ratio between the anionic NPs and the cationic PEs. Here, the acido-basic titration was used to evaluate the number of available electrostatic charges per particle (see Additional file 1: SI-2).

For the 8.3-nm *γ*-Fe_2_O_3_ NPs coated by PAA_2K_, we got the number of carboxylate groups available per particle $N^{{\mathrm{COO}}^{-}}=12,500$. We can then calculate the total number of the negative charges $N^{{}^{-}}$ in the stock solution by:

(2) $$N^{{}^{-}}=\frac{V_{\mathrm{NP}}\times c_{\mathrm{NP}}}{M_{W}^{\mathrm{NP}}}\times N_{A}\times N^{\mathrm{COO}-}$$

Where *V*_NP_ and *c*_NP_ are the volume and mass concentration, respectively, of the stock solution containing NPs; $M_{W}^{\mathrm{NP}}$ is the molecular weight of the 8.3-nm *γ*-Fe_2_O_3_ NPs; *N*_*A*_ is the Avogadro constant.

For the cationic polymers, we calculated the number of positive charges $N^{{}^{+}}$ from their molecular structures.

(3) $$N^{{}^{+}}=\frac{V_{\mathrm{poly}}\times c_{\mathrm{poly}}}{M_{W}^{\mathrm{poly}}}\times N_{A}\times \frac{M_{W}^{\mathrm{poly}}}{M_{W}^{\mathrm{monomer}}}\times n$$

Where *V*_poly_ and *c*_poly_ are the volume and mass concentration, respectively, of the polymer stock solution; $M_{W}^{\mathrm{monomer}}$ and $M_{W}^{\mathrm{poly}}$ are the molecular weight of the monomer and of the polymer, respectively; *n* is the number of the positive charges per each monomer. In this work, the two stock solutions were always prepared at the same concentration: *c*_NP_ = *c*_poly_. We took the average molecular weight of particle $M_{W}^{\mathrm{NP}}$ = $M_{W}^{\mathrm{SLS}}$ = 5.82 × 10^6^ g mol^−1^ which was measured as a function of the concentration by using static light scattering (see Additional file 1: SI-2). Thus particles-polymer charges ratio *Z* can be expressed as:

(4) $$Z=\frac{N^{-}}{N^{+}}=X\times \frac{12,500\times M_{W}^{\mathrm{monomer}}}{5.82\times 10^{6}\times n}$$

By using Equation 4, we can then easily control the charges ratio *Z* by tuning the particle to polymer volume ratio *X* = *V*_NP_*/V*_poly_. For the four different polymers mentioned above, the relations between *Z* and *X* were shown in Table 2.

**Table 2:** **Particles-polymer charges ratio *Z*** **(*X*) of the mixing solution containing these PEs and magnetic NPs**

**Polymer**	***M***_**w**_**(g mol**^**−1**^**)**	***n***	***Z *** **( *X *)**
PTEA_11K_-*b*-PAM_30K_	44,400	1	1.9 *X*
PDADMAC	100,000	1	0.35 *X*
PEI	2,000	1	0.26 *X*
PAH	15,000	4	0.2 *X*

Where *n* is the number of the positive charges per each monomer.

The direct mixing procedure was preferred to titration experiments because it allowed to explore a broad range in mixing ratios (*Z =* 10^−3^ to 100) and simultaneously to keep the total concentration in the dilute regime [40]. As far as the kinetics is concerned, the formation of the aggregates occurred very rapidly on mixing, i.e., within a time scale inferior to 1 s for both copolymer and homopolymers. In the ranges investigated, the dispersions resulting from direct mixing were fully reproducible.

#### Dilution

In the dilution process, deionized water was added to mixtures of PAA_2K_-coated nanoparticles and PEs (PTEA_11K_-*b*-PAM_30K_ copolymer or HomopPEs) stepwise, changing *I*_*S*_ from 3 to 5 × 10^−2^ M. In this process, the overall concentration was decreased by a factor of 60. Since the aggregates formed by dilution are much larger than the unassociated polymer and particles, the measurements of their hydrodynamic properties up to the lowest ionic strength could be easily fulfilled. The critical ionic strength of the transition noted $I_{S}^{c}$is defined in the ‘Results and discussion’ section.

#### Dialysis

Mixtures of PAA_2K_-coated NPs and PEs in the presence of 3 M of NH_4_Cl were dialyzed against deionized water at pH 7 using a Slide-a-Lyzer® cassette, Rockford, IL, USA, with MWCO of 10 kD cutoff membrane (Thermo Scientific, Waltham, MA, USA). In the protocol of the dialysis [51,65], adopted strategy involved in a first step is the preparation of two separate NH_4_Cl solutions containing respectively the (i) the anionic iron oxide NPs and (ii) the cationic polymer. In a second step, the two solutions were mixed with each other and it was checked by dynamic light scattering that the two components remained dispersed. In a third step, the ionic strength of the mixture was progressively diminished by dialysis. The volume of the dialysis bath was 300 times larger than that of the samples. The electrical conductivity of the dialysis bath was measured during the ion exchange and served to monitor the desalting kinetics [51]. In the condition described here, the whole process reached a stationary and final state within 50 to 100 min. Once the ionic strength of the bath reached its stationary value, typically 10^−3^M, the dispersions inside the dialysis membrane were studied by optical microscopy. The dialysis experiment between the initial and final ionic strengths was characterized by an average rate of ionic strength change *dI*_*S*_*/dt* ~ −10^−4^m s^−1^. Note that with dialysis, the NPs and PEs concentration remained practically constant.

### Optical microscopy and transmission electron microscopy

For optical microscopy, phase-contrast images of the magnetic wires were acquired on an IX71 inverted microscope (Olympus, Shinjuku-ku, Japan) equipped with × 20 and × 40 objectives. Dispersion (2 μl) at concentration 0.01 wt.% was deposited on a glass plate and sealed into to a Gene Frame® (Abgene/Advanced Biotech, Totowa, NJ, USA) dual adhesive system. We used a EXi Blue camera (QImaging, Surrey, BC, Canada) and Metaview software (Universal Imaging Inc., Brandywine, PA, USA) as acquisition system. In order to determine the length distribution of the wires, pictures were digitized and treated by the ImageJ software (http://rsbweb.nih.gov/ij/).

TEM was carried out on a JEOL-100 CX microscope, Akishima-shi, Japan, at the SIARE facility of University Pierre et Marie Curie (Paris 6). TEM was used to characterize both the individual PAA_2K_ coated *γ*-Fe_2_O_3_ NPs (magnification × 160,000) and the NPs/PEs aggregates (magnification from × 10,000 to × 100,000).

### Light scattering and electrophoretic mobility

Static and dynamic light scattering were monitored on a Brookhaven spectrometer (BI-9000AT autocorrelator, Brookhaven, GA, USA) for measurements of the Rayleigh ratio *R*(*q*,*c*) and of the collective diffusion constant *D*(*c*). We measured the electrophoretic mobility and zeta potential versus *Z* for aggregates formed from NPs and PEs by using Zeatsizer Nano ZS Malvern Instrument at PECSA, University Pierre et Marie Curie (Paris 6), Paris, France). The Rayleigh ratio was obtained from the scattered intensity *I*(*q*,*c*) measured at the wave-vector *q* according to [66]

(6) $$R\left(q,c\right)=R_{\mathrm{std}}\frac{I\left(q,c\right)-I_{\mathrm{Water}}}{I_{\mathrm{Tol}}}{\left(\frac{n}{n_{\mathrm{Tol}}}\right)}^{2}$$

Here, *R* and *n*_Tol_ are the standard Rayleigh ratio and refractive index of toluene, respectively, *I*_Water_ and *I*_Tol_ are the intensities measured for the solvent and for the toluene in the same scattering configuration and *q =* (4*πn*/*λ*) sin(*θ*/2) (*n* being the refractive index of the solution and *θ* the scattering angle), respectively. In this study, the Rayleigh ratio *R*(*q*,*c*) was measured as a function of the mixing ratio *Z* and for the different desalting kinetics. With the Brookhaven spectrometer, the scattering angle was *θ* = 90°, whereas for the NanoZS, it was *θ =* 173°, corresponding to wave-vectors *q =* 1.87 × 10^−3^ Å^−1^ and *q* = 2.64 × 10^−3^ Å^−1^, respectively. In quasi-elastic light scattering, the collective diffusion coefficient *D*_0_was measured in the dilute concentration range (*c =* 0.1 wt.%). The hydrodynamic diameter of the colloids was calculated according to the Stokes-Einstein relation, *D*_*H*_ *= k*_*B*_*T/3πηD*_*0*_, where *k*_*B*_ is the Boltzmann constant, *T* is the temperature (*T* = 298 K), and *η* is the solvent viscosity (0.89 × 10^−3^ Pa s). The autocorrelation functions of the scattered light were interpreted using both the method of cumulants and the CONTIN fitting procedure provided by the instrument software.

## Results and discussion

### Direct mixing

**Figure 3:**
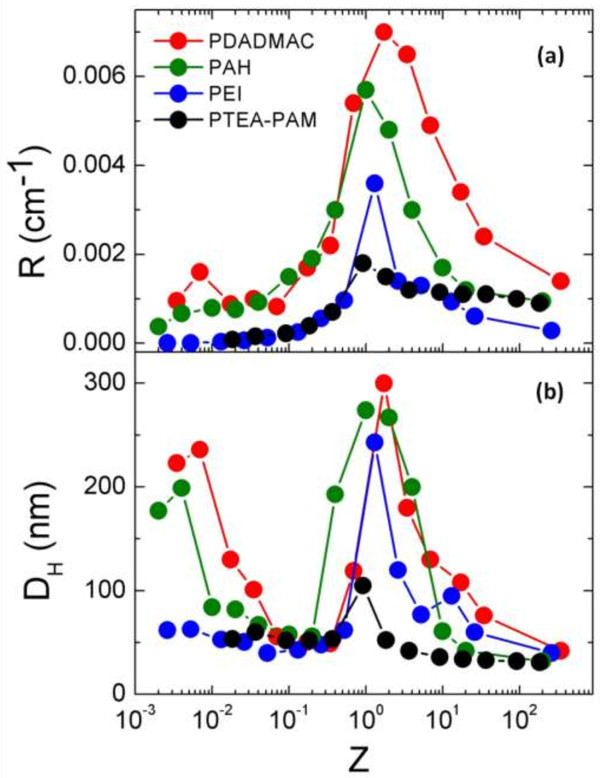
**Rayleigh ratios *R*(*q*,*c*) and hydrodynamic diameters (*D***_***H***_**) obtained for PAA**_**2K**_**-*γ*-Fe**_**2**_**O**_**3 **_**complexed with PTEA**_**11K**_**-*b*-PAM**_**30K **_**copolymers. (a)** Normalized Rayleigh ratios *R*(*X*)/*R*∞ obtained at *q =*1.87 × 10^−3^Å^−1^for *γ*-Fe_2_O_3_-PAA_2K_ complexed directly with copolymers and homoPEs: PTEA_11K_-*b*-PAM_30K_ (black closed symbols), PDADMAC (red closed symbols), PEI (blue closed symbols), and PAH (green closed symbols), for the NPs-PEs charges ratio*Z*ranging from 10^−3^to 100. The total concentration is *c* ~ 0.1 wt.% and temperature *T* ~ 25°C. **(b)** Hydrodynamic diameter *D*_*H*_ as a function of *Z* for the same system.

Figure 3 displays the Rayleigh ratios *R*(*q*,*c*) and hydrodynamic diameters (*D*_*H*_) obtained for PAA_2K_-*γ*-Fe_2_O_3_ complexed with PTEA_11K_-*b*-PAM_30K_ copolymers, PDADMAC, PAH, and PEI respectively, for *Z* ranging from 10^−3^ to 100, at *T* = 25°C. For both copolymers and homoPEs, *R*(*q*,*c*) and *D*_*H*_ were found to pass through a sharp maximum at isoelectric point (*Z* = 1), indicating a maximum aggregation between oppositely charged particles and polymers. Over the whole *Z* range in Figure 3, the data for PTEA_11K_-*b*-PAM_30K_ diblocks (*D*_*H*_ = 100 nm) were lower than those of the homoPEs (*D*_*H*_ = 300 nm), indicating a slowed complexation or aggregation process when non-interacting neutral blocks are present in solution. The sharp and intense maximum at *Z* = 1 was found to be similar with the polyelectrolyte-liposome aggregation, which were reported by Cametti et al. [55-58], which suggest that they have similar aggregation mechanism: by adding increased quantities of the polyion, with the progressive neutralization of the absorbed particles, the size of the aggregates initially increases. At the stochiometry condition, when the overall charge of the polyion equals the overall charge at the particle surface, the size of the aggregates reaches its maximum value. Beyond this point, their size decreases again when the polyion is in large excess. This behavior can be explained by considering that, beyond the isoelectric condition, the polyion which is added in excess to the suspension, keeps adsorbing onto the particle surface. In this way, on the two sides of the isoelectric point (for *Z* > 0.3 and *Z* > 7), when the charge of the adsorbed polyions exceeds or falls short of the original charge of the particle by similar amounts, the resulted aggregates have similar sizes (approximately 100 nm) and are stable for few weeks. It can be explained that, on the two sides near the border of the ‘destabilization zone’, the electrostatic repulsion induced by the extra polymers (*Z* > 0.3) or particle charges (*Z* > 7) can slow and soften their aggregation process. Theses long-lived stable clusters state obtained at the two sides of isoelectric point was often called ‘arrested states’.

### Dilution

**Figure 4:**
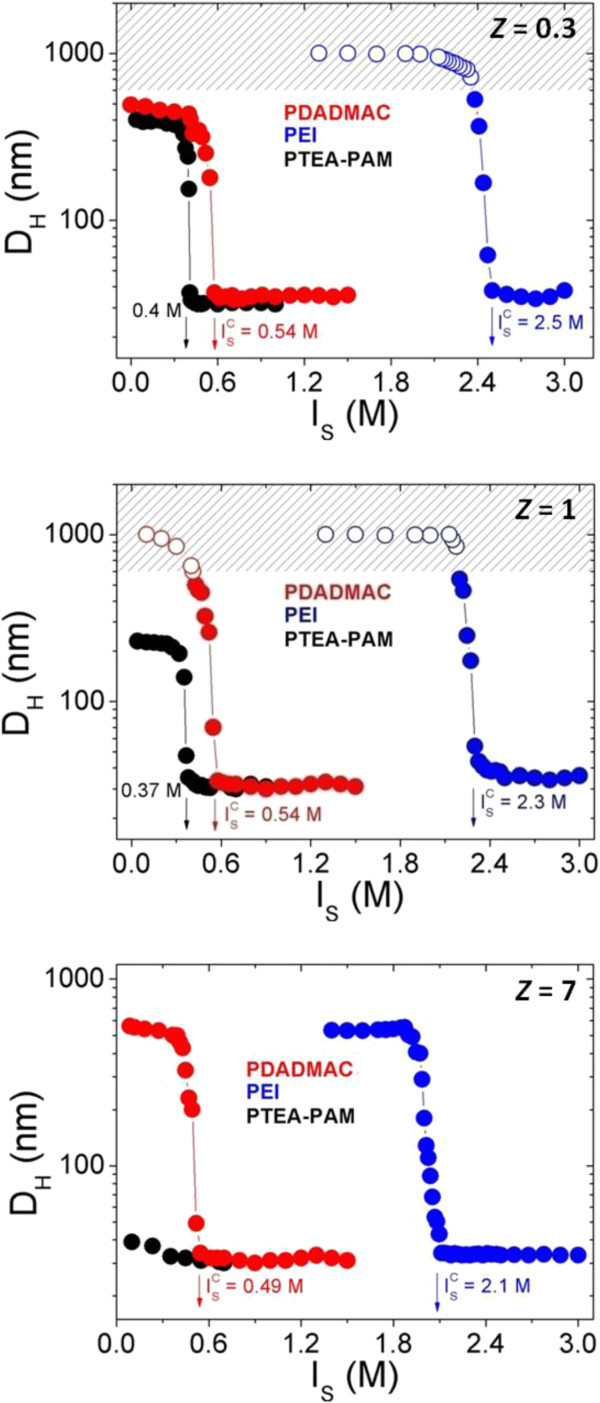
***D***_***H ***_**versus *I***_***S ***_**during the dilution process.** Ionic strength dependence of the hydrodynamic diameter *D*_*H*_ for a dispersion containing *γ*-Fe_2_O_3_-PAA_2K_ particles and oppositely charged PTEA_11K_-*b*-PAM_30K_ (black closed symbols), PDADMAC (red closed symbols), and PEI (blue closed symbols) at *Z* = 0.3, *Z* = 1, and *Z* = 7. At *Z* = 1, with decreasing *I*_*S*_, an abrupt transition was observed at a critical ionic strength at 0.38 ± 0.01 M, 0.54 ± 0.01 M, and 2.3 ± 0.01 M for the solution containing PTEA_11K_-*b*-PAM_30K_, PDADMAC, and PEI, respectively. At *Z* = 0.3 and *Z* = 7, their critical ionic strength was found to be 0.40 ± 0.01 M, 0.54 ± 0.01 M, 2.5 ± 0.01 M, 0.49 ± 0.01 M, and 2.1 ± 0.01 M respectively. At *Z* = 1, because of their maximum complexation, the size of clusters based on PDADMAC and PEI are superior to 1 μm at the end of dilution, which induced a macroscopic phase separation (marked by the empty symbols and patterned area).

**Figure 5:**
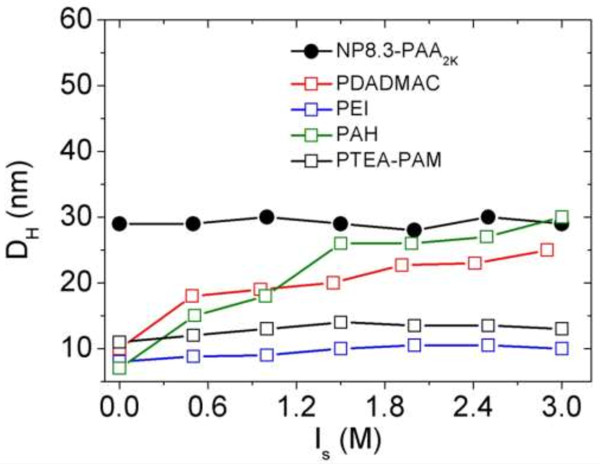
**Ionic strength dependence of the hydrodynamic diameter *D***_***H***_**for a dispersion containing the individual components.** Which is PAA_2K_-coated *γ*-Fe_2_O_3_ nanoparticles (closed symbols), PTEA_11K_-*b*-PAM_30K_ (black open circles), PDADMAC (red open squares), PEI (blue open squares), and PAH (green open squares).

From the results in the preceding paragraph, we find that the direct mixing method is not ideal since it cannot control both size and morphology of resulted aggregates. Recently, we have developed an original method to control the complexation of NPs and copolymers PTEA_11K_-*b*-PAM_30K_ at isoelectric point (*Z* = 1). The protocols consisted of two steps. The first step was based on the screening of the electrostatic interactions by bringing the dispersions to 1 M of salt. In the second step, the salt was removed progressively by dialysis or by dilution. Using this controllable desalting method without and with an external magnetic field, we got spherical clusters of 200 nm and straight wires of 10 to 100 μm, respectively [48,50,51]. In the experiments of dilution, DI water was added stepwise to particles/polymers salted dispersion with 3 M NH_4_Cl and the hydrodynamic diameter were determined by light scattering. Figure 4 shows the *D*_*H*_ versus *I*_*S*_ during the dilution process. For the dispersion prepared at isoelectric point (*Z* = 1), an abrupt transition was observed at a critical ionic strength $I_{S}^{c}$ = 0.38 ± 0.01 M, 0.54 ± 0.01 M, and 2.3 ± 0.01 M for PTEA_11K_-*b*-PAM_30K_, PDADMAC, and PEI, respectively. This transition illustrates two different colloidal states of the dispersion during the dilution process: above $I_{S}^{c}$, the particles and polymers remain independent and unaggregated; below $I_{S}^{c}$, the anionic particles are retained within dense and spherical clusters, thanks to the cationic polymer ‘glue’. Dispersions prepared apart from the isoelectric point, i.e., at *Z* = 0.3 and *Z* = 7 were found to undergo similar desalting transitions. The critical ionic strengths $I_{S}^{c}$ corresponding to the different polymer and different particles-polymers charges ratio *Z* were shown in Table 3. As a comparison, Figure 5 displays ionic strength dependence of the hydrodynamic diameter *D*_*H*_ for a dispersion containing only the individual components, which is PAA_2K_-coated *γ*-Fe_2_O_3_ nanoparticles, PTEA_11K_-*b*-PAM_30K_, PDADMAC, PEI, and PAH. These individual components are all stable up to an *I*_*S*_ of 3 M, and no transition could be evidenced.

**Table 3:** **Critical ionic strength**$I_{S}^{c}$** obtained at the different particles-polymers charges ration *Z***

**Polymer**	$I_{S}^{c}$**at *Z*** **= 0.3 (M)**	$I_{S}^{c}$**at *Z*** **= 1.0 (M)**	$I_{S}^{c}$**at *Z*** **= 7 (M)**
PTEA_11K_-*b*-PAM_30K_	0.40 ± 0.01	0.38 ± 0.01	-
PDADMAC	0.54 ± 0.01	0.54 ± 0.01	0.49 ± 0.01
PEI	2.5 ± 0.01	2.3 ± 0.01	2.1 ± 0.01

**Figure 6:**
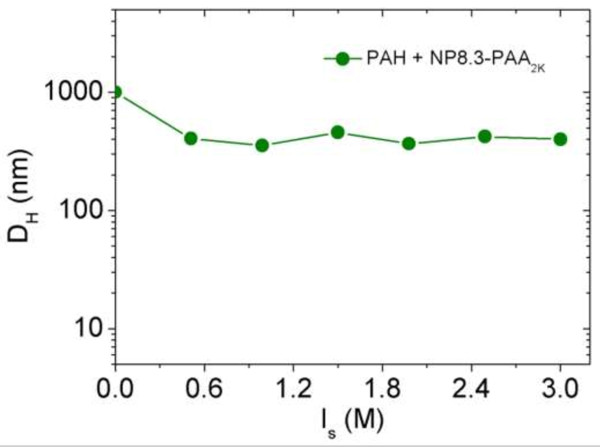
**Ionic strength dependence of the hydrodynamic diameter *D***_***H***_**.** For a dispersion containing *γ*-Fe_2_O_3_-PAA_2K_ particles and oppositely charged PAH polymers. With decreasing *I*_*S*_, no abrupt transition was observed.

For a dispersion containing NPs and PTEA_11K_-*b*-PAM_30K_ at *Z* = 1, because of their non-interacting neutral blocks, the *D*_*H*_ of resulted clusters was limited at 200 nm at the end of dilution process. Equally at the isoelectric point, the size of clusters based on the homoPEs (PDADMAC and PEI) increased however rapidly below the critical ionic strength $I_{S}^{c}$. At the end of dilution, the size of the large aggregates is superior to 1 μm and the aggregates sediment at the bottom of tube, which suggests that the interactions are stronger with homoPEs than with the diblock. For the dispersions prepared from homoPEs at *Z* = 0.3 and *Z* = 7, we found the clusters of smaller sizes (*D*_*H*_ ~ 500 nm) and we did not find a sedimentation until the end of dilution process. These results confirmed the existence of ‘arrested state’ at the two sides of ioelectric point. In this work, the desalting transition was shown to be a general process for homoPEs. The effective screening was found for PDADMAC and PEI but not for PAH. For this later system, even at 3 M, the oppositely charged species interacted strongly and large aggregates were formed (*D*_*H*_ = 400 nm, shown in Figure 6).

### Dialysis

**Figure 7:**
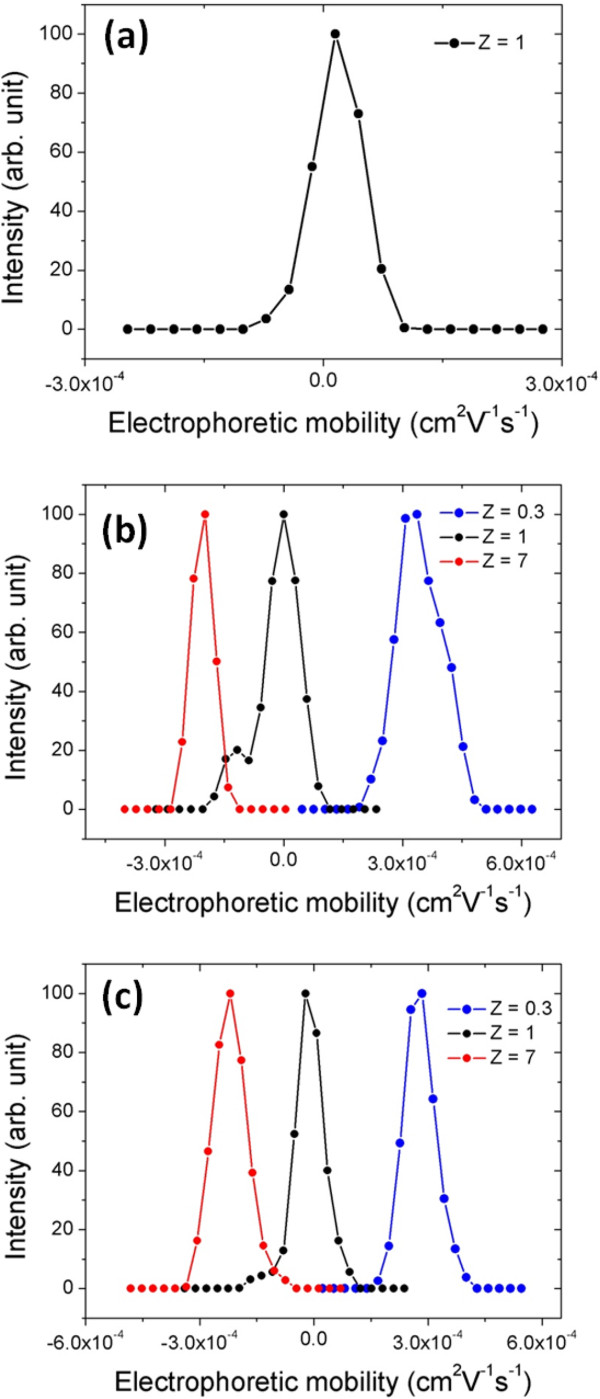
**Intensity *versus *electrophoretic mobility.** For *γ*-Fe_2_O_3_-PAA_2K_/PTEA_11K_-*b*-PAM_30K_**(a)**, *γ*-Fe_2_O_3_-PAA_2K_/PDADMAC **(b)**, and *γ*-Fe_2_O_3_-PAA_2K_/PEI **(c)** clusters obtained by dialysis without the presence of external magnetic field.

Since the effective screening effects were evidenced for PDADMAC and PEI, we then investigate the dialysis process of PDADMAC/NPs and PEI/NPs salted dispersion at *Z* = 0.3, *Z* = 1, and *Z* = 7. In fact, dialysis and dilution experiments are both based on the same desalting procedure. During the dialysis, the NPs and polymers are kept inside the membrane (10 KD MWCO) of the dialysis cassette. After 1 h of dialysis, we obtained spherical clusters formed by PDADMAC and by PEI, respectively. Their hydrodynamic diameters *D*_*H*_, determined by using Zetasizer Nano ZS Malvern Instrument, were in good agreement with the results obtained in dilution experiments (see Table 4). Moreover, we anticipate that the clusters made with an excess of polymers should be positively charged and those with an excess of nanoparticles negatively charged, while the clusters obtained at isoelectric point should be neutral. In this work, electrokinetic measurements were performed on these cluster dispersions to determine their electrophoretic mobility *μ*_*E*_ and *ζ*-potential (shown in Table 4). The intensities distributions of *μ*_*E*_ are shown in Figure 7. At *Z* = 0.3 and 7, *μ*_*E*_ is centered around +3 × 10^−4^ cm^2^ V^−1^ s^−1^ and −2.1 × 10^−4^ cm^2^ V^−1^ s^−1^, respectively for both PDADMAC and PEI. At Z = 1, *μ*_*E*_ is approximately 0 for both copolymer and homoPEs. For PDADMAC and PEI, their intensity distribution of *μ*_*E*_ (Figure 7b,c) clearly showed a charge inversion of the resulted clusters, passing from negative values (at *Z* = 7) to neutral charges (at *Z* = 1), then pass to negative value (at *Z* = 0.3).

**Table 4:** **Values of the electrophoretic mobilities *μ***_***E ***_**and *ζ*-potentials**

**Polymer used to form the clusters**	***D***_***H ***_**(nm)**	***μ***_***E ***_**(cm**^**2**^**/V s)**	***ζ *** **(mv)**
PTEA_11K_-*b*-PAM_30K_			
*Z* = 1	200	0.18 × 10^−4^	2.3
PDADMAC			
*Z* = 0.3	500	2.79 × 10^−4^	35.6
*Z* = 1	1,000	−0.12 × 10^−4^	−1.6
*Z* = 7	550	−2.20 × 10^−4^	−28
PEI			
*Z* = 0.3	1,000	3.43 × 10^−4^	43.8
*Z* = 1	1,000	−0.16 × 10^−4^	−2.0
*Z* = 7	550	−2.05 × 10^−4^	−26

For clusters made from PTEA_11K_-b-PAM_30K_, PDADMA, C and PEI polymers and oppositely charged nanoparticles. The electrophoretic mobility intensities are shown in Figure 7.

### Dialysis under the application of magnetic field

**Figure 8:**
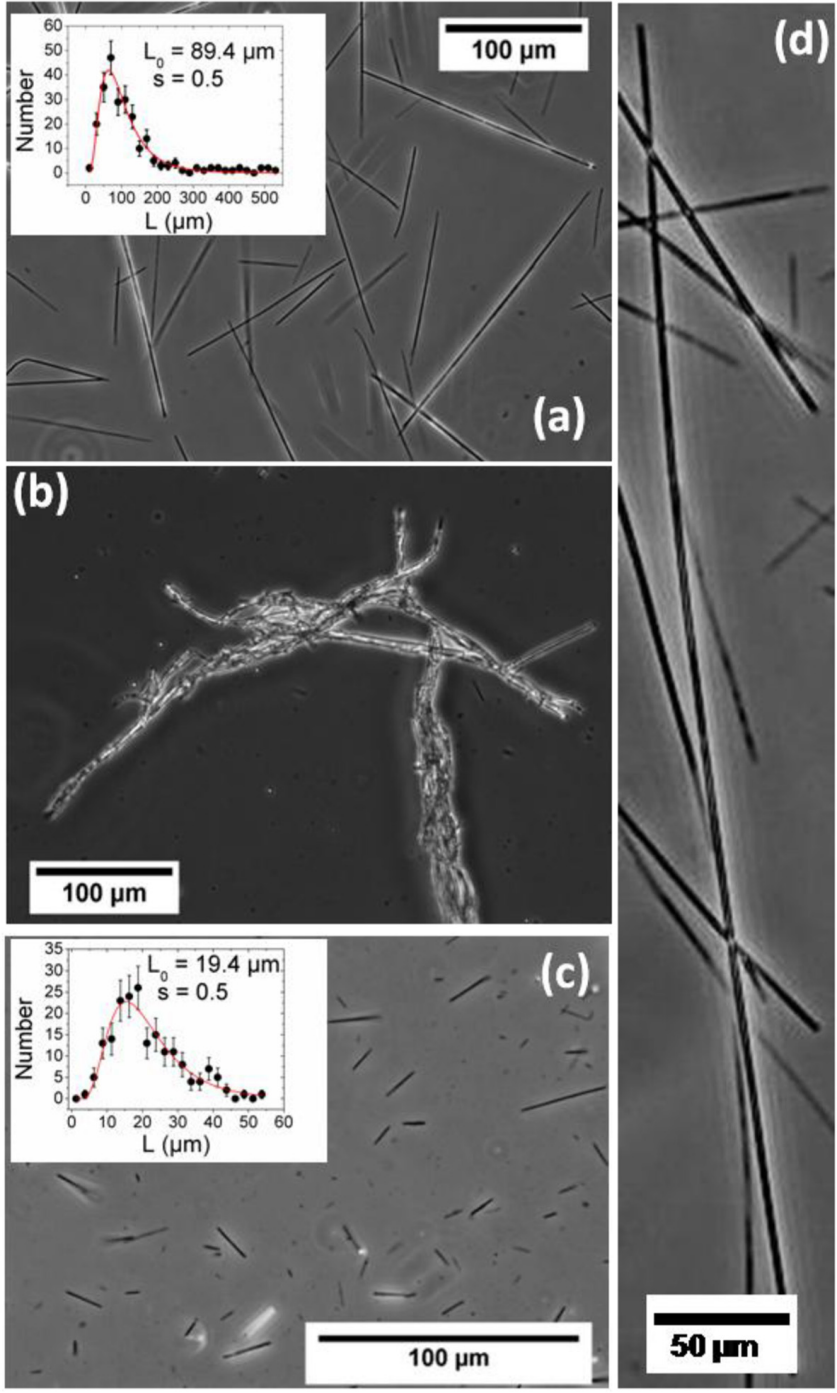
**Phase-contrast optical microscopy images (×10, ×20, and × 40) of a dispersion of nanostructured wires.** The wires are made from 8.3 nm *γ*-Fe_2_O_3_ particles and PDADMAC at *Z* = 0.3 **(a)**, *Z* = 1 **(b)**, and *Z* = 7 **(c)**. At *Z* = 0.3, we could get the wires with maximum length of 500 μm (0.5 mm) directly by the particles of 8.3 nm (d). Length distribution of wires was shown in insert. The continuous line was derived from best fit calculation using a log-normal distribution.

Then, we investigate the dialysis with the presence of an external magnetic field of 0.3 T for the same dispersions in order to generate one-dimensional growth of magnetic wires [51,65]. Figure 8 displays the optical transmission microscopy images of aggregates made of PDADMAC and PAA_2K_-*γ*-Fe_2_O_3_ dispersions at Z = 0.3 (Figure 8a), 1 (Figure 8b), and 7 (Figure 8c). Large and irregular aggregates in the 100-μm range were obtained at *Z* = 1. This result showed that, at the isoelectric point and without the presence of non-interacting neutral blocks, the PDADMAC/PAA_2K_–*γ*-Fe_2_O_3_ interactions were strong and their electrostatic complexation cannot be controlled. However, dialysis with an extra polymer charges (*Z* = 0.3) or an extra particle charges (*Z* = 7) resulted straight wires with the regular forms. These straight and regular wires illustrate that, at arrested states and with the presence of extra polymer or particle charges, the PDADMAC/PAA_2K_-*γ*-Fe_2_O_3_ interactions can be softened and thus their one-dimensional aggregation can be controlled. Series of images similar to that of Figure 8a,c were analyzed quantitatively to retrieve the wires length distribution. In both cases, the length distribution was found to be well accounted for by a log-normal function of the form:

(7) $$p\left(L,L_{0},s_{L}\right)=\frac{1}{\sqrt{2\pi }\beta_{L}\left(s_{L}\right)L}\exp\left(-\frac{\ln^{2}\left(L/L_{0}\right)}{2\beta_{L}{\left(s_{L}\right)}^{2}}\right)$$

**Figure 9:**
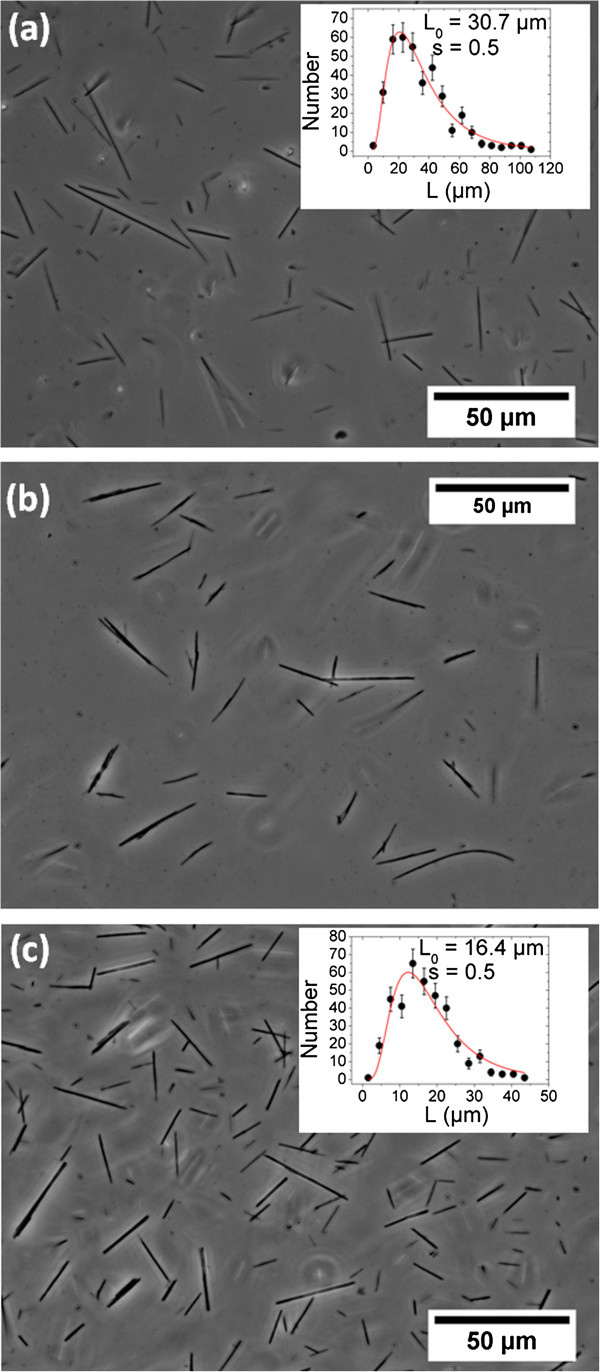
**Phase-contrast optical microscopy images (×10, ×20, and × 40) of a dispersion of nanostructured wires.** The wires are made from 8.3 nm γ-Fe_2_O_3_ particles and PEI at *Z* = 0.3 **(a)**, *Z* = 1 **(b)**, and *Z* = 7 **(c)**. Length distribution of wires was shown in insert. The continuous line was derived from best fit calculation using a log-normal distribution.

Where *L*_*0*_ is defined as the median length and *β*_*L*_*(s*_*L*_*)* is related to the polydispersity index *s*_*L*_ by the relationship $\beta_{L}\left(s\right)=\sqrt{\ln\left(1+s_{L}^{2}\right)}$. The polydispersity index is defined as the ratio between the standard deviation (〈*L*^2^〉 − 〈*L*〉^2^)^1/2^ and the average length 〈*L*〉. For wires made from PDADMAC at *Z* = 0.3 and Z = 7, one obtained *L*_*0*_ = 90 ± 3 and 19 ± 1 μm, respectively. The polydispersity *s*_*L*_ was similar for the two specimens and equal to 0.5 (see inserts in Figure 9). It is also interesting to note that at *Z* = 0.3, we obtained few wires with maximum length of 500 μm (0.5 mm) directly by the particles of 8.3 nm (Figure 8d). The study on the dialysis of PEI/PAA_2K_-*γ*-Fe_2_O_3_ dispersion presented same results like PDADMAC (Figure 9): we got straight and regular wires at *Z* = 0.3 with *L*_*0*_ = 31 ± 1 μm and at *Z* = 7 with *L*_*0*_ = 16 ± 1 μm. These results showed that the wire formation is a general phenomenon that does not depend on the nature of the polycations.

**Figure 10:**
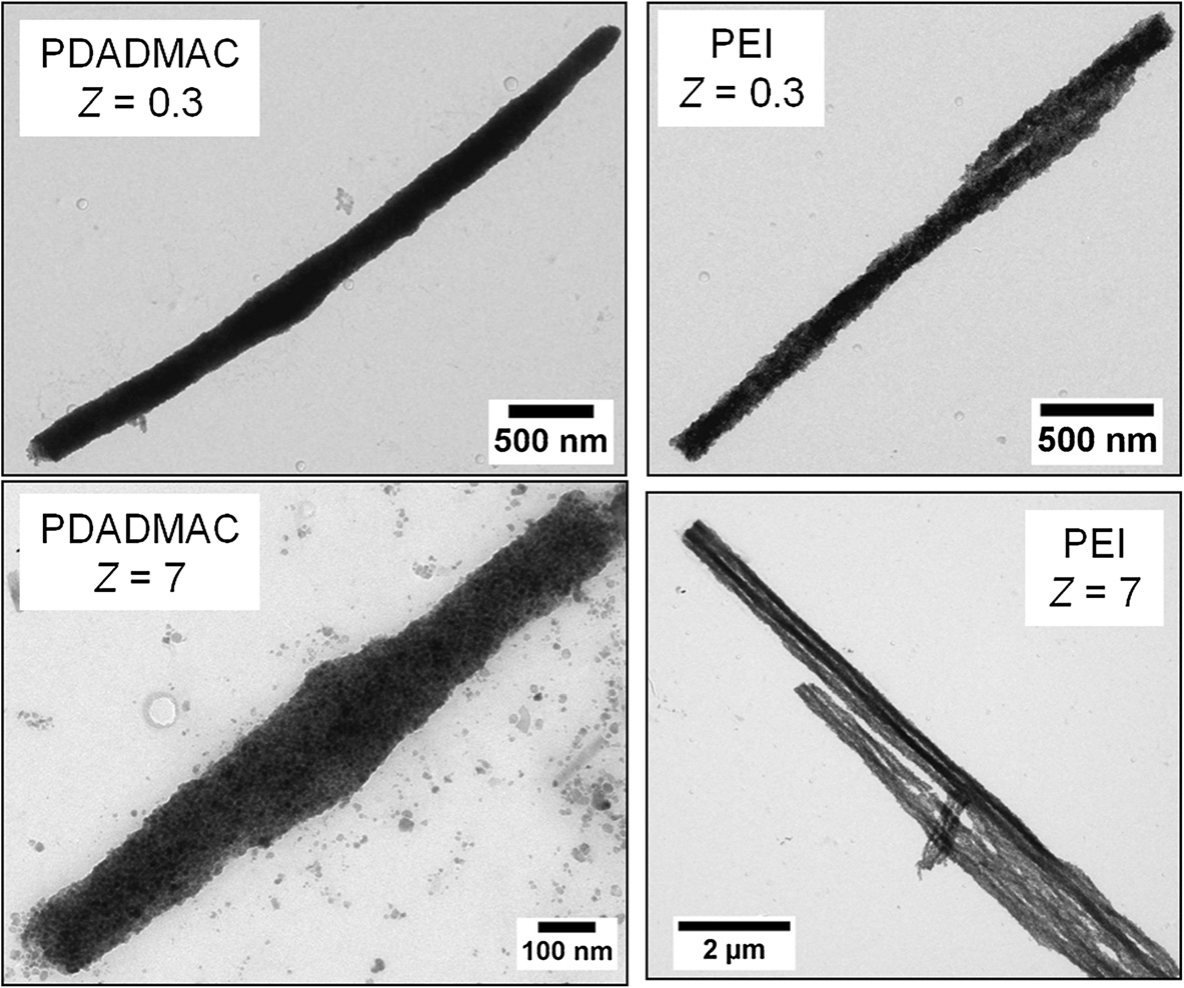
**TEM images of wires obtained at *Z*** **= 0.3 and *Z*** **= 7.**

In order to reveal the microscopic structure of these straight and regular wires, TEM was performed on their dilute dispersions (at concentration 0.01 wt.%). Figure 10 displayed elongated bodies with diameters comprised between 150 and 400 nm of the magnetic wires made of PDADMAC and of PEI. From these figures, we find that the individual particles held together with similar particles densities and formed the elongated core structure.

**Figure 11:**
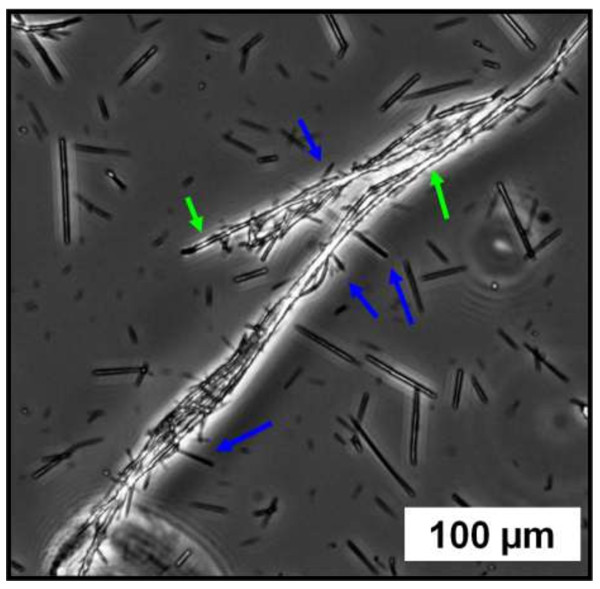
**Phase-contrast optical microscopy images (×20).** Of a dispersion containing the direct mixing of the rods formed from PDADMAC at *Z* = 0.3 and Z = 7. The attachment of the short and negatively charged rods (obtained at *Z* = 7 and green arrows) onto the long and positively charged rods (obtained at *Z* = 0.3 and blue arrows) confirmed an evident electrostatic attraction.

From our previous work, we concluded that the mechanism of magnetic wires proceeds in two steps: (i) the formation and growth of spherical clusters of particles and (ii) the alignment of the clusters induced by the magnetic dipolar interactions [51]. For the kinetics, the cluster growth and their alignment occurred in parallel, leading to a continuous welding of the cylindrical structure. From the results of clusters shown in Table 4 and Figure 7, we can thus conclude that the magnetic wires made at *Z* = 0.3 should be positively charged and those at Z = 7 negatively charged. To further confirm it, long (*L*_0_ = 89.4 μm) and positively charged PDADMAC wires were mixed directly with short (*L*_0_ = 19.4 μm) and negatively charged PDADMAC wires. The turbidity of the suspension was increased revealing the formation of larger brush-like aggregates (Figure 11), where the short wires agglutinated onto the larger ones, thanks to attractive electrostatic interactions. Same aggregation between oppositely charged PEI wires was also evidenced by optic microscopy (see Additional file 1: SI-4).

### Reorientation behavior under the application of magnetic field

These PDADMAC and PEI formed wires exhibited the same mechanical and magnetic properties as the ones made from PTEA_11K_*-b-*PAM_30K_ copolymers: they were all rigid aggregates with superparamagnetic properties inherited from the single particles [48,51].

## Conclusions

In conclusion, we have studied the electrostatic complexation between cationic homoPEs and anionic PAA_2K_-*γ*-Fe_2_O_3_. The complexation was realized by using three different approaches such as direct mixing, dilution, and dialysis. In the first method, named direct mixing, we mixed directly the stock polymer and NPs solutions without salt added. The mixing of the two initial solutions was characterized by the particle-polymer charges ratio *Z*. By using DLS, we confirmed the existences of a ‘destabilization state’ for the dispersion prepared at isoelectric point (*Z* = 1) and ‘long-lived stable clusters state’ (arrested states) for the ones prepared apart from isoelectric point (*Z* = 0.3 and Z = 7). The dilution of salted solution (with 3 M NH_4_Cl) containing NPs and homoPEs confirmed that there also exists a screen effect for widespread homoPEs (PDADMAC and PEI) as the copolymer.

We then investigated the dialysis of these salted dispersions of under an external magnetic field (*B* = 0.3 T) in order to produce one-dimensional magnetic wires. At isoelectric point, we obtained large aggregates of 100 μm with irregular morphologies, indicating the strong attractive interaction between homoPEs with charged NPs and their uncontrolled complexation. At *Z* = 0.3 and Z = 7, the straight and regular magnetic wires were obtained, indicating that the extra polymer or particle charges can soften their strong attractive interaction. These wires can be either positively (obtained at *Z* = 0.3) or negatively (obtained at *Z* = 7) charged on surface. These homoPEs formed wires, as the wires made from PTEA_11K_*-b-*PAM_30K_ copolymers, were rigid aggregates with superparamagnetic properties inherited from the single particles. We thus have shown that the previous copolymer-based co-assembly strategy could be generalized to strong and weak polyelectrolytes. In terms of cost and practicality, this represents a remarkable improvement. Beyond, the evident surface charges induced by the amine or carboxyl functions can not only enhance their colloidal stability but also facilitate their future functionalization. This simple and general approach opens significant perspectives for the design of multifunctional hybrid materials.

## Abbreviations

DLS: dynamic light scattering; homoPEs: homopolyelectrolytes; NPs: nanoparticles; PAH: poly(allylamine hychloride); PDADMAC: poly(diallyldimethylammonium chloride); PEI: poly(ethyleneimine); PEs: polyelectrolytes; PTEA11K-b-PAM30K: poly (trimethylammonium ethylacrylate)-b-poly (acrylamide); TEM: transmission electron microscopy; VSM: vibrating sample magnetometry.

## Competing interests

The authors declare that they have no competing interests.

## Authors' contributions

MY designed the whole study, carried out the electrostatic complexation between NPs and homoPEs, analyzed the data, and wrote the manuscript. LQ and JF synthesized NPs, did the organic coating around bare NPs, and participated in the complexation between NPs and homoPEs. YR participated in the design of the study and coordination. All authors read and approved the final manuscript.

## Supplementary Material

Additional file 1 **Supporting information.** SI-1. Characterization of nanoparticle sizes and size distribution. SI-1.1.Vibrating sample magnetometry (VSM). SI-1.2. Transmission Electron Microscopy (TEM). SI-1.3. Dynamic Light Scattering (DLS). SI-2. Characterization of polymer coated nanoparticle. SI-2.1. Number of poly (acrylic acid) chains per particle. SI-2.2. Number of electrostatic charges borne by the PAA2K-coated particles. SI-3. Mixture of the oppositely charged wires of PEI. Click here for file
