# Supplementary material for: Electrostatic complexation of polyelectrolyte and magnetic nanoparticles: from wild clustering to controllable magnetic wires
Source: Nanoscale Res Lett. 2014 May 1;9(1):198. doi: 10.1186/1556-276X-9-198 (PMC4029954; doi:10.1186/1556-276X-9-198)
Supplement: Additional file 1 — Supporting information. SI-1. Characterization of nanoparticle sizes and size distribution. SI-1.1.Vibrating sample magnetometry (VSM). SI-1.2. Transmission Electron Microscopy (TEM). SI-1.3. Dynamic Light Scattering (DLS). SI-2. Characterization of polymer coated nanoparticle. SI-2.1. Number of poly (acrylic acid) chains per particle. SI-2.2. Number of electrostatic charges borne by the PAA2K-coated particles. SI-3. Mixture of the oppositely charged wires of PEI. [file 1556-276X-9-198-S1.doc]

**Supporting information**

Electrostatic complexation of polyelectrolyte and magnetic nanoparticles: from wild clustering to controllable magnetic wires

**Minhao Yan*, Li Qu, Jiangxia Fan and Yong Ren**

*Outline*

**SI-1 – Characterization of nanoparticle sizes and size distribution**

**SI-1.1 –** Vibrating sample magnetometry (VSM)

**SI-1.2 –** Transmission Electron Microscopy (TEM)

**SI-1.3 –** Dynamic Light Scattering (DLS)

**SI-2 – Characterization of polymer coated nanoparticle**

**SI-2.1 –** Number of poly (acrylic acid) chains per particle

**SI-2.2 –** Number of electrostatic charges borne by the PAA2K-coated particles

**SI-3 – Mixture of the oppositely charged wires of PEI**

**SI-1 – Characterization of nanoparticle sizes and size distribution**

*SI-1.1 – Vibrating sample magnetometry (VSM)*

Vibrating sample magnetometry (VSM) consisted in measuring the magnetization *versus* excitation *M(H)* for a solution at volume fraction *ϕ* from the signal induced in detection coils when the sample is moved periodically in an applied magnetic field (thanks to synchronous detection and with an appropriate calibration). Here we used the superparamagnetic NPs having dominant populations at 8.3 nm. Fig. S1 shows the evolution of the macroscopic magnetization *M(H)* normalized by its saturation value *M*_S_ for the γ-Fe_2_O_3_ superparamagnetic NP. Here, *M*_S_ = *ϕ m*_S_, where *m*_S_ is the specific magnetization of colloidal maghemite (m_S_ = 3.5×10^5^ A m^−1^) which is lower than for bulk maghemite and decrease when the diameters of the superparamagnetic NP decrease due to some disorder of the magnetic moments located near the surface. The solid curve in Fig. S1 was obtained using the Langevin function for superparamagnetism convoluted with a log-normal distribution function of the particle size. The parameters of the distribution are the median diameter ($D_{0}^{VSM}$ = 8*.*3 ± 0*.*1 nm) and the polydispersity ($s^{VSM}$ = 0*.*21 ± 0*.*02).


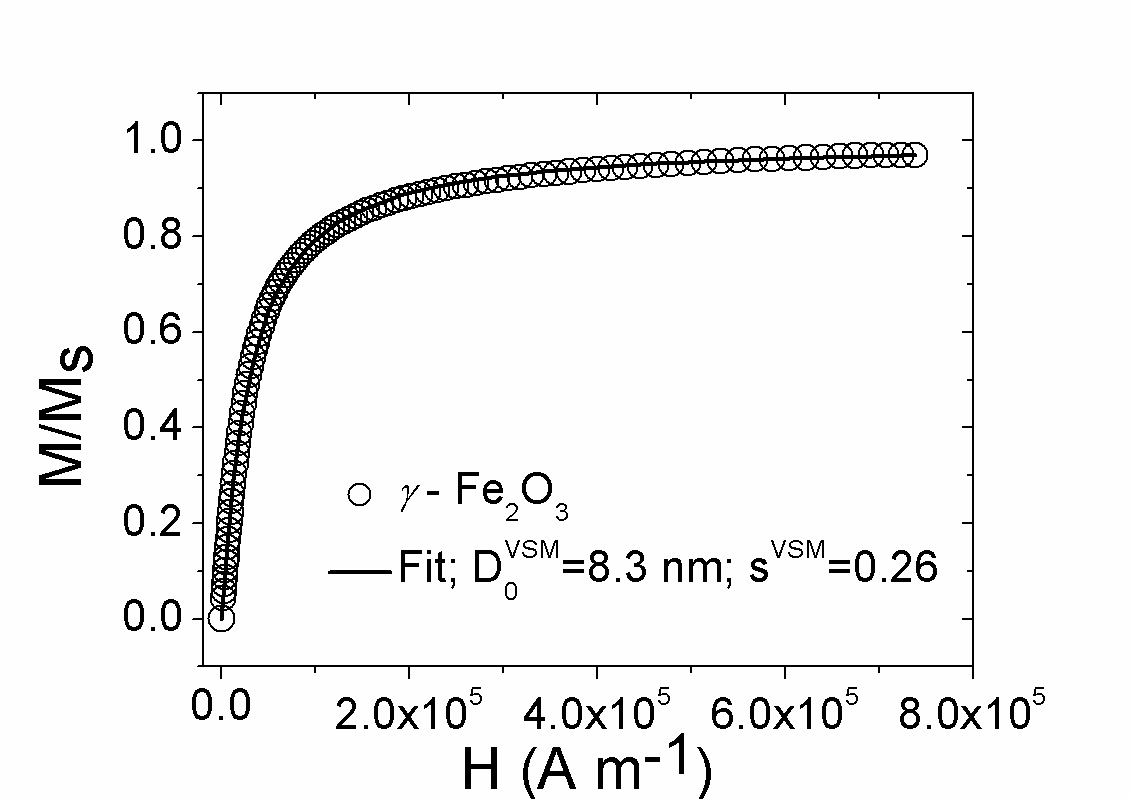


**Figure S1 :** Magnetic field dependence of the macroscopic magnetization *M(H)* normalized by its saturation value *M*_S_ for cationic maghemite dispersions. The solid curve was obtained using the Langevin function for superparamagnetism convoluted with a log-normal distribution function for the particle sizes, given with median diameters $D_{0}^{VSM}$ and polydispersity $s^{VSM}$.

*SI-1.2 – Transmission Electron Microscopy (TEM)*

In fact, the median diameter $D_{0}^{VSM}$ obtained by VSM is due to the crystal structure inside the *γ*-Fe_2_O_3_ nanoparticle. We then compared these values with the physical diameters $D_{0}^{TEM}$by using the transmission electron microscopy (TEM). Fig. S2 displays image of the *γ*-Fe_2_O_3_ superparamagnetic NPs.


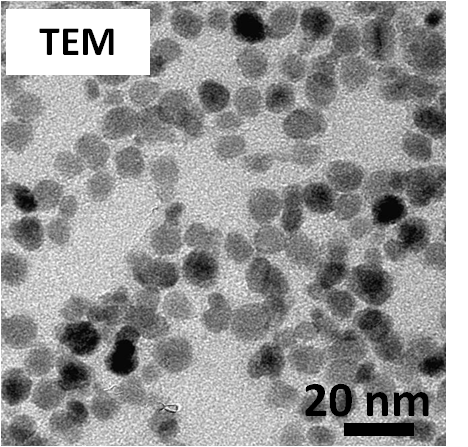


**Figure S2 :** Iron oxide superparamagnetic NPs as observed by TEM. The stability of the dispersion was ensured by electrostatic interactions mediated by the native cationic charges.

Probability distribution functions of size for these superparamagnetic NPs observed by TEM on a series of images similar to that of Fig. S2 were shown in Fig. S3. The data could be fitted using a log-normal function with physical diameter $D_{0}^{TEM}$= 9*.*3 ± 0*.*2 nm and polydispersity $s^{TEM}$ = 0.18 ± 0.01.

$p(D,D_{0},s_{D})=\frac{1}{\sqrt{2\pi}\beta_{D}(s_{D})D}\exp\left(-\frac{\ln^{2}(D/D_{0})}{2\beta_{D}(s_{D})^{2}}\right)$ SI(1)

These values were found to be in relative good agreement with the ones obtained from VSM, albeit with a minor difference between the median diameter $D_{0}^{VSM}$ and physical diameter$D_{0}^{TEM}$, which could originate from defects located close to the particle surfaces, and that would not contribute to magnetic properties.


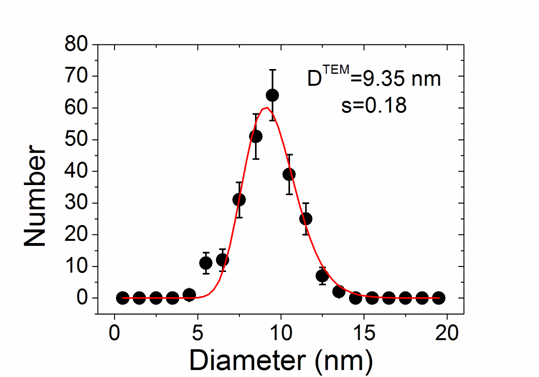


**Figure S3 :** Probability distributions function of size for the *γ*-Fe_2_O_3_ superparamagnetic nanoparticles. The continuous line was derived from best fit calculation using a log-normal distribution. For these dispersions, the average size of the superparamagnetic NPs were found to be 9.3 nm, and the polydispersity 0.18.

**SI-2 – Characterization of polymer coated nanoparticle**

*SI-2.1* ***–*** *Number of poly (acrylic acid) chains per particle*

Static light scattering has been utilized to determine the molecular weight of the PAA_2K_-coated nanoparticles and hence to derive the number *n_ads_* of adsorbed polymers per particle. To do so, 200 mL of a PAA_2K_–*γ*-Fe_2_O_3_ (at 8.3 nm) solutions have been prepared at 0.4 wt. %, and pH 10. Fig. S4 displays the Rayleigh ratios *RR*(*c*) of the PAA-coated particles as function of the *γ*-Fe_2_O_3_ weight concentration in the range 0-0.4 wt. %. This scattered intensity is compared to that of the bare particles. It was also checked that the hydrodynamic diameter of the sols (bare and coated) remains unchanged over the dilution range. In the two cases, *RR*(*c*) varies linearly with *c* according to

$RR(c)=KcM_{w}^{app}$ (1)

where $M_{w}^{app}$ is the weight-average apparent molecular weight of the scattering entities and $k=4\pi n^{2}\left(dn/dc\right)^{2}/N_{A}\lambda^{4}$is the scattering contrast (*N*_A_ is the Avogadro number and *dn/dc* is the refractive index increment, measured on a Chromatix KMX-16 differential refractometer).

For the bare NPs, the Rayleigh ratio measured as a function of the concentration yielded a weight average molecular weight M_W_ = 5.82×10^6^ g mol^-1^.

The ratio between the two straight lines in Fig. S4 is 1.18. This ratio can be calculated as function the characteristics of the two systems, yielding

$\frac{RR_{coated NP}\left(c\right)/c}{RR_{bare NP}\left(c\right)/c}= \frac{K_{coated NP}}{K_{bare NP}}\left(1+n_{ads}\frac{M_{W}^{Pol}}{M_{W}^{NP}}\right)^{2}$ (2)

Here we assume that the scattering contrasts *K* are identical for the bare and coated particles. From the comparison between the data of Figure 1 and eq 2, we find $N_{ads}^{PAA_{2K}}$= 258 ± 25, corresponding to 0.95 polymer chain per nm^2^. This sample and rapid method can be used to determinate quantitatively the amount of the coating materials around the small colloidal objects.

**
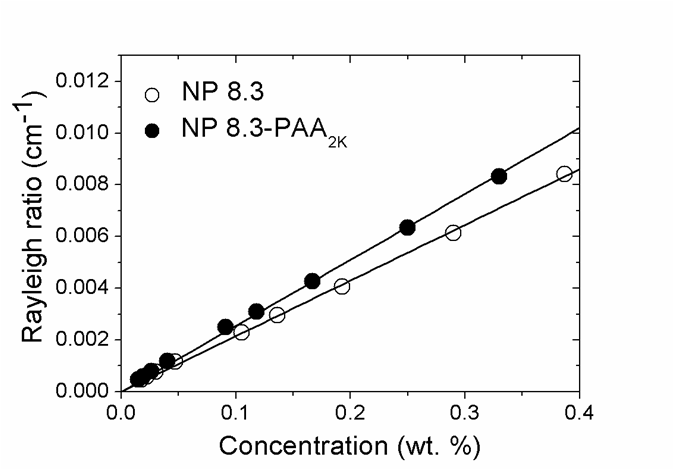
**

**Figure S4 :** Rayleigh ratios measured by static light scattering for bare γ-Fe2O3 particles (at 8 nm) at pH 2 and for PAA_2K_–*γ*-Fe_2_O_3_ solutions at pH 10. The concentration in abscise is that of the bare particles. These data allow us to derive the number of adsorbed polymers per particle $N_{ads}^{PAA_{2K}}$= 258 ± 5 (from eq 2).

*SI-2.2* ***–*** *Number of electrostatic charges borne by the PAA_2K_-coated particles*

The acido-basic titration is used to evaluate the number of electrostatic charges available per particle. The black curves of the Fig. S5 exhibit the evolution of pH as a function of the added NaOH whereas the bleu curves represent the calculation of the derivative of pH with respect to the number of mol of NaOH added. In this figure, three equivalent points corresponding to the three maxima exhibited in the derivative of the pH versus n(NaOH) curves are observed and indicated by the dashed line 1, 2 and 3. The pH of the dispersions was first decreased to pH 2 by adding HCl. The first maximum of the derivative (dashed line 1 on the Figure 2) corresponds to equivalent point of the titration of this HCl and occurs just above pH 4. The acido-basic titration done on the poly (acrylic acid) of Mw = 2100 g/mol in the same conditions as described before reveals an equivalent point at pH 9.5 and a pKa (PAA_2K_) = 6.14. Thus, the position of the equivalence point corresponding to the titration of the carboxylic acid groups of the PAA_2K_ adsorbed to the nanoparticles was identified to occur at a pH slightly above 8 and is indicated by the dashed line 2 on the Fig. S5. The difference of amount of NaOH added between the first and the second equivalence points is equal the amount of carboxylic acid function titrated, and is denoted as “n_eq_”. The value of the pH at 1/2 “n_eq_” is the pKa of the PAA_2k_ adsorbed chains and is denoted pKa (NP-PAA_2K_). The pKa (NP-PAA_2K_) value of 6.73, averaged from the measurements of the (a), (b) and (c) experiments of the Fig. S5, is slightly lower than pKa (PAA_2K_) = 6.14, indicating a more basic condition of the carboxylate groups of the PAA_2K_ chains adsorbed to the NP than those of “free” PAA_2K_ chains. This could be interpreted as a consequence of a higher density of carboxylic acid groups when polyelectrolyte chains are geometrically constrained as in the conformation they should have in the NP-PAA_2k_ system, compared to their chemical environment in a solution of PAA_2k_ in the dilute regime. Appearing at pH 10.3, the third maximum of the derivative (dashed line 3) evidences the acido-basic properties of the ammonium ions that were used in the redispersion process.

From the number of carboxylate groups titrated, n_eq_, the average density of carboxylic acid groups on each particle can be valued. The number of nanoparticles is calculated as $n\left(NP\right)=m_{\gamma-Fe_{2}O_{3}}/M_{W}^{TEM}$, the mass of *γ*-Fe_2_O_3_ divided by M_W_^TEM^, the average molecular weight of the nanoparticles. So on, the number of titrated carboxylate groups per NP can be estimated as n_eq_, the number of carboxylic acid groups, divided by the number of nanoparticles. For the PAA_2K_ coated NPs, 12500 carboxylate groups per NP were titrated.

**
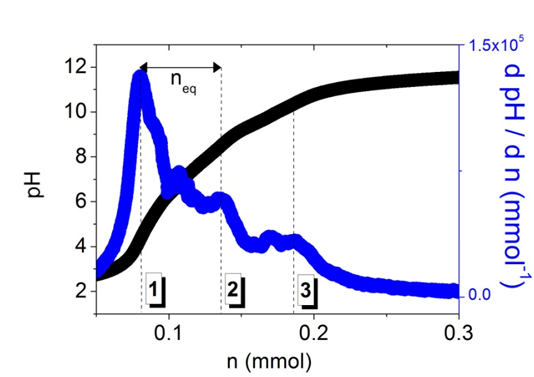
**

**Figure S5 :** Acido-basic Titrations of *γ*-Fe_2_O_3_-PAA_2K_ dispersions by a NaOH 0.0357 M solution injected at 0.01 mL / min under Nitrogen atmosphere: 6.141 g of PAA_2k_ coated NPs at 0.42 wt% in iron oxide. The black curve represents the pH measured in function of the number of mol of NaOH, whereas the blue curve represents the calculation of the derivative of pH with respect to the number of mol of NaOH. “n_eq_” means the equivalent quantite of carboxylate titrated

**SI-3 – Mixture of the oppositely charged wires of PEI**

Similar like the rods of PDADMAC, the wires of PEI obtained at the two sides of the isoelectric point should also have the clear net charges: positive charges for *Z* = 0.3 and negative for *Z* = 7. To confirm their opposite charges, we mixed directly these two kinds of wires together. Fig. S6 shows an optical transmission microscopy image of the mixing wires. The attachment of the short and negatively charged wires (obtained at *Z* = 7) onto the long and positively charged wires (obtained at *Z* = 0.3) confirmed an evident electrostatic attraction.

**
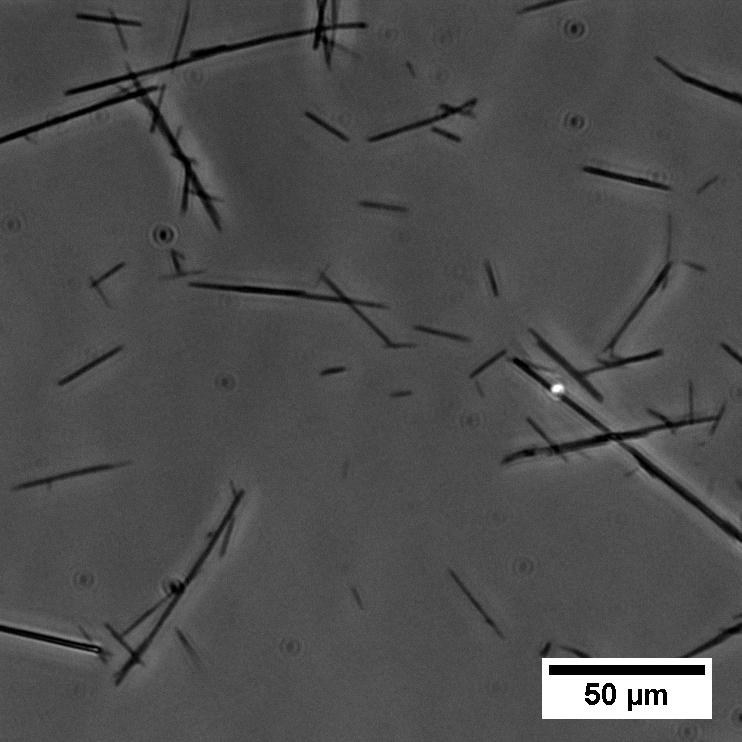
**

**Figures S6 :** Phase-contrast optical microscopy images (20×) of a dispersion containing the direct mixing of the rods formed from PEI at *Z* = 0.3 and Z = 7. The attachment of the short and negatively charged rods (obtained at *Z* = 7) onto the long and positively charged rods (obtained at *Z* = 0.3) confirmed an evident electrostatic attraction between them.
